# Comparison of efficiency between i-gel blind intubation and i-gel-assisted bronchoscopic intubation during cardiopulmonary resuscitation: randomized simulation study

**DOI:** 10.1186/2197-425X-3-S1-A547

**Published:** 2015-10-01

**Authors:** W Kim, HY Choi, YS Jang, GH Kang

**Affiliations:** Emergency Medicine, Hallym University Kangnam Sacred Heart Hospital, Seoul, Republic of Korea

## Introduction

i-gel has been used as a conduit for blind and bronchoscopic operative intubation. However, it is not easy that conventional polyvinyl chloride (PVC) tracheal tube pass through i-gel in emergent intubation during cardiopulmonary resuscitation. We considered wire-reinforced silicone tube could pass through i-gel more easily than conventional PVC tube.

## Objectives

This study aimed to compare intubation performances among i-gel blind intubation (IGI), i-gel bronchoscopic intubation (IBRI) and intubation using Macintosh laryngoscope (MCL) applying two kinds of endotracheal tube during chest compressions. We hypothesized that IGI using wire-reinforced silicone tube could achieve tracheal intubation most rapidly and successfully.

## Methods

In 23 emergency physicians, a prospective randomized crossover study was conducted to examine the three intubation techniques using two kinds of endotracheal tube. Primary outcomes were the intubation time. Secondary outcomes were the cumulative success rate for intubation.

## Results

The mean intubation time using IGI was shorter (*p* < 0.017) than that of IBRI and MCL in both endotracheal tubes (17.6 vs. 29.3 vs. 20.2 in conventional PVC tube; 14.6 vs. 27.4 vs. 19.9 in wire-reinforced silicone tube; sec). There were no significant (*p* > 0.05) differences between conventional PVC and wire-reinforced silicone tube for each intubation technique. The cumulative success rate using IGI was also shorter (*p* < 0.017) than that of IBRI and MCL in both endotracheal tubes.

## Conclusions

IGI could be an effective intubation technique in emergent intubation during cardiopulmonary resuscitation.
